# Deep Learning Hybrid Method to Automatically Diagnose Periodontal Bone Loss and Stage Periodontitis

**DOI:** 10.1038/s41598-020-64509-z

**Published:** 2020-05-05

**Authors:** Hyuk-Joon Chang, Sang-Jeong Lee, Tae-Hoon Yong, Nan-Young Shin, Bong-Geun Jang, Jo-Eun Kim, Kyung-Hoe Huh, Sam-Sun Lee, Min-Suk Heo, Soon-Chul Choi, Tae-Il Kim, Won-Jin Yi

**Affiliations:** 1 Department of Oral and Maxillofacial Radiology, School of Dentistry and Dental Research Institute, Seoul National University, Seoul, Korea; 2 Department of Biomedical Radiation Sciences, Graduate School of Convergence Science and Technology, Seoul National University, Seoul, Korea; 3 Department of Periodontology, School of Dentistry and Dental Research Institute, Seoul National University, Seoul, Korea; 4 Department of Oral and Maxillofacial Radiology, Seoul National University Dental Hospital, Seoul, Korea

**Keywords:** Biomedical engineering, Periodontitis

## Abstract

We developed an automatic method for staging periodontitis on dental panoramic radiographs using the deep learning hybrid method. A novel hybrid framework was proposed to automatically detect and classify the periodontal bone loss of each individual tooth. The framework is a hybrid of deep learning architecture for detection and conventional CAD processing for classification. Deep learning was used to detect the radiographic bone level (or the CEJ level) as a simple structure for the whole jaw on panoramic radiographs. Next, the percentage rate analysis of the radiographic bone loss combined the tooth long-axis with the periodontal bone and CEJ levels. Using the percentage rate, we could automatically classify the periodontal bone loss. This classification was used for periodontitis staging according to the new criteria proposed at the 2017 World Workshop on the Classification of Periodontal and Peri-Implant Diseases and Conditions. The Pearson correlation coefficient of the automatic method with the diagnoses by radiologists was **0.73** overall for the whole jaw (p < 0.01), and the intraclass correlation value **0.91** overall for the whole jaw (p < 0.01). The novel hybrid framework that combined deep learning architecture and the conventional CAD approach demonstrated high accuracy and excellent reliability in the automatic diagnosis of periodontal bone loss and staging of periodontitis.

## Introduction

Periodontal diseases, including gingivitis and periodontitis, are some of the most common diseases that mankind faces. Periodontitis is the 6th most prevalent disease worldwide. It leads to alveolar bone loss, tooth loss, edentulism, and masticatory dysfunction, which indirectly affects nutrition^1^. Periodontitis also impairs quality of life and self-esteem, which imposes huge socio-economic impacts and healthcare costs^1,2^. The classification of periodontitis has been repeatedly modified in an attempt to align it with the emerging scientific evidence in the last 30 years^3^. In 2017, the American Academy of Periodontology and the European Federation of Periodontology provided a new definition and classification framework for periodontitis based on a multidimensional staging and grading system^4^. The staging is related to the severity and extent of periodontitis at present. The grading adds another dimension, and allows the rate of progression to be considered^4^. Clinically, the periodontal health can be evaluated just by measuring the clinical attachment loss (CAL) using probing pocket depths and gingival recession. However, this method is limited in its reliability related to the probing force, angulation, placement, and tip diameter^5–7^. Radiographic bone loss (RBL) should be used if the CAL is not available^4^.

Computer-aided diagnosis (CAD) has been used to identify cavities and periodontitis lesions, as well as maxillary sinusitis, osteoporosis, and other pathologies in the oral and maxillofacial field^8^. It can provide dental professionals with a valuable second opinion by automatically detecting and classifying pathological changes. Recently, computer aided diagnoses (CAD) based on deep learning have been used extensively for solving complex problems in radiology^9^. However, studies of deep learning applications have been limited in the field of oral and maxillofacial imaging. The deep learning method has been applied to: detect landmarks in cephalograms^10^; detect teeth and classification^11–13^; diagnose cavities^14–18^; and detect maxillary sinusitis^19^.

To date, there have only been a few studies that have investigated the use of deep learning in the diagnosis of periodontitis of the jaw. The convolution neural network (CNN) was used in the detection of periodontally compromised teeth on intraoral radiographs^20^. CNN-based methods were also proposed for detecting radiographic bone loss (RBL) on dental panoramic radiographs^21,22^. However, these methods only detected the region that showed RBL, and could not quantify or classify it in order to stage the periodontitis. As far as we know, no previous studies have diagnosed periodontal bone loss quantitatively or automatically in order to stage the periodontitis on dental panoramic radiographs. Therefore, the aim of this study was to develop an automated method for diagnosing periodontal bone loss (of individual teeth) for staging the periodontitis on dental panoramic radiographs using the deep learning hybrid method for the first time. We proposed a novel hybrid framework of deep learning architecture and the conventional CAD approach to detect and classify periodontal bone loss according to the 2017 World Workshop criteria^4^.

## Results

### Detection performance for the periodontal bone level, the CEJ level, and the teeth by the CNN

**Figure 1:**
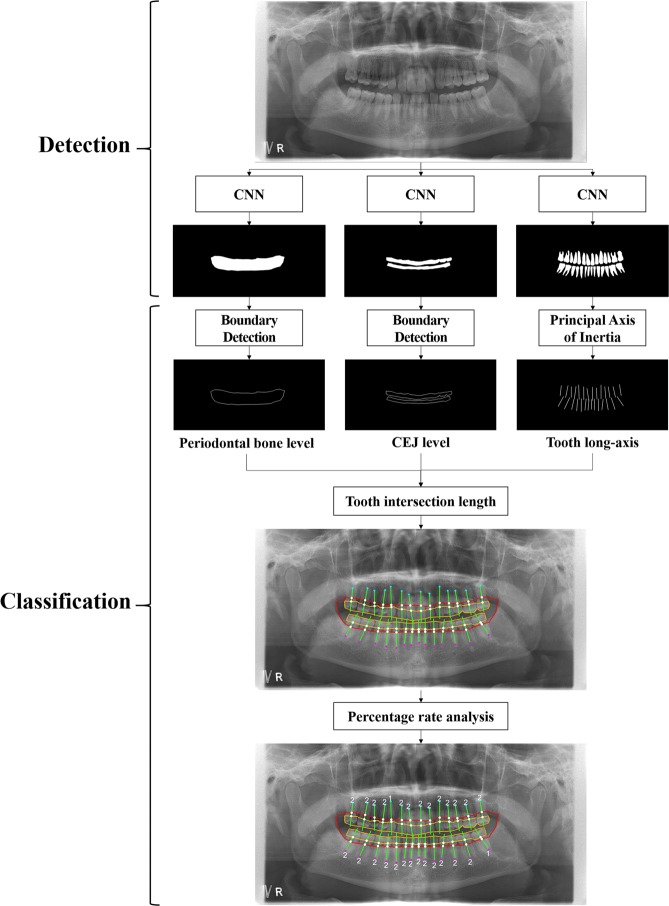
Overall procedure for a hybrid framework of deep learning architecture and the conventional CAD approach to detect and classify periodontal bone loss.

**Figure 2:**
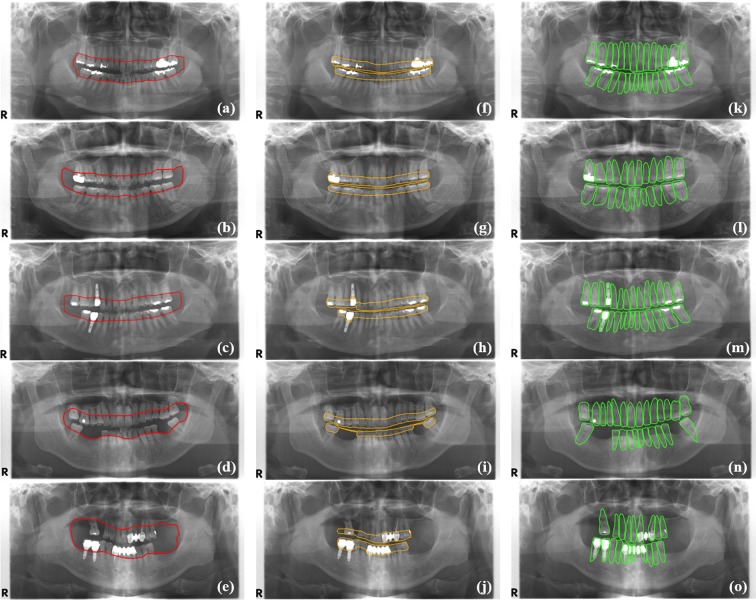
Detection results for the periodontal bone level (**a**–**e**), the CEJ level (**f**–**j**), and the teeth and implants (**k**–**o**) by the developed CNN.

Figure 1 shows the overall procedure for a hybrid framework of deep learning architecture and the conventional CAD processing to automatically detect and classify periodontal bone loss. Figure 2 shows the detection results for the periodontal bone level, the cementoenamel junction (CEJ) level, and the teeth and implants using the developed CNN. The Jaccard index, the pixel accuracy (PA) and dice coefficient values were 0.92, 0.93, and 0.88, respectively, for the detection of the periodontal bone level using CCN. These values were 0.87, 0.91, and 0.84, respectively, for the CEJ level, and 0.87, 0.91, and 0.83, respectively, for the teeth and implants (Table 1).

**Table 1:** PA, dice coefficient, and Jaccard index for detection performance of the periodontal bone level, the CEJ level, and the teeth by the developed CNN.

	Pixel Accuracy	Dice coefficient	Jaccard index
Periodontal Bone level	0.92 ± 0.03	0.93 ± 0.02	0.88 ± 0.03
CEJ level	0.87 ± 0.04	0.91 ± 0.02	0.84 ± 0.04
Teeth	0.87 ± 0.06	0.91 ± 0.01	0.83 ± 0.02

### Classification performance of the mean absolute difference between stages

**Figure 3:**
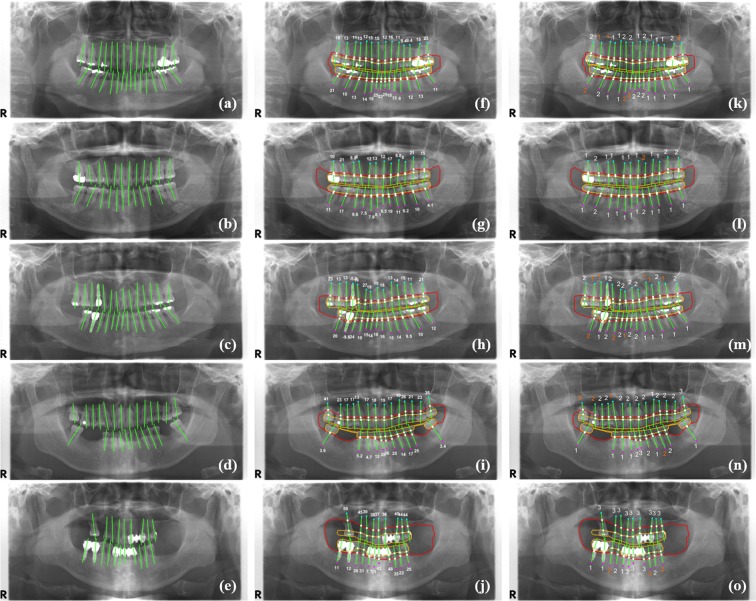
The long-axis orientations of the tooth and the implant (**a**–**e**), the intersection points of the tooth (implant) long-axis with the periodontal bone level and the CEJ level (fixture top level), the percentage rate of the radiographic bone loss (**f**–**j**), and the stages of the periodontitis for each tooth and implant (**k**–**o**) (correctly classified stages in white color, and incorrectly classified stages in orange color).

Figure 3 shows the long-axis orientations of the tooth and the implant determined by the principal axes of inertia, and the intersection points of the tooth (implant) long-axis with the periodontal bone level and the CEJ level (fixture top level). Figure 3 also shows the percentage rate of the radiographic bone loss (RBL), and the classified stages of the periodontitis for teeth and implants according to the new criteria proposed at the 2017 World Workshop on the Classification of Periodontal and Peri-implant Diseases and Conditions^4^. In order to evaluate the classification performance of the periodontal bone loss, the mean absolute differences (MAD) between the stages classified by the automatic method and diagnosed by the professor, fellow, and resident’s diagnoses were compared (Fig. 3). These MAD values were 0.21, 0.25, and 0.25 for the professor, fellow, and resident, respectively, for the teeth of the whole jaw (Table 2). The MAD for the incisors, canine, premolars, and molars was 0.26 ± 0.44, 0.21 ± 0.43, 0.21 ± 0.42, and 0.32 ± 0.48, respectively. The MAD for the incisors and molars was higher than those of the canine and premolars (p > 0.05). The overall MAD between stages by the automatic method and by all of the radiologists was **0.25**. The stages classified by the automatic method were not significantly different from those diagnosed by the radiologists with regard to the maxillary, mandibular, and whole jaw (p > 0.05).

**Table 2:** The mean absolute differences between periodontitis stages obtained using the automatic method and those diagnosed by the radiologists (a professor, a fellow and a resident) (*p > 0.05).

	Maxilla	Mandible	Whole jaw
Professor	0.23 ± 0.19*	0.19 ± 0.12*	0.21 ± 0.12*
Fellow	0.26 ± 0.17*	0.25 ± 0.13*	0.25 ± 0.11*
Resident	0.29 ± 0.10*	0.21 ± 0.11*	0.25 ± 0.07*
Mean	0.27 ± 0.45*	0.23 ± 0.43*	0.25 ± 0.44*

### Classification performance of correlations between stages

The Pearson correlation coefficients (PCC) of the automatic method with the professor, fellow, and resident’s diagnoses were 0.76, 0.73 and 0.70, respectively, for the whole jaw (p < 0.01) (Table 3). The PCC between the automatic method and the professor’s diagnosis showed the highest correlation. In addition, the overall PCC value of the developed method using the radiologists’ diagnoses was **0.73** (p < 0.01). This PCC showed a strong correlation between the radiologists and the automatic diagnoses. The intraclass correlation coefficient (ICC) values of the automatic method and the professor, fellow, and resident’s diagnoses were 0.86. 0.84, and 0.82, respectively, for the whole jaw (p < 0.01) (Table 4). The ICC between the automatic method and the professor’s diagnosis showed the highest correlation. The automatic classification in the developed method showed the high reliability with the radiologists’ diagnoses. The overall ICC value between the developed method and the radiologists’ diagnoses was 0.91 (p < 0.01). This ICC value indicates excellent reliability of the automatic diagnoses for periodontal bone loss.

**Table 3:** The Pearson correlation coefficients (PCC) between stages obtained using the automatic method and those diagnosed by the radiologists (a professor, a fellow and a resident) (*p < 0.01).

	Automatic method	Professor	Fellow	Resident
Automatic method	1	0.76*	0.73*	0.70*
Professor	0.76*	1	0.72*	0.70*
Fellow	0.73*	0.72*	1	0.70*
Resident	0.70*	0.70*	0.70*	1

**Table 4:** The intraclass correlation coefficient (ICC) between stages obtained using the automatic method and those diagnosed by radiologists (a professor, a fellow and a resident) (*p < 0.01).

	Automatic method	Professor	Fellow	Resident
Automatic method	1	0.86*	0.84*	0.82*
Professor	0.86*	1	0.84*	0.82*
Fellow	0.84*	0.84*	1	0.82*
Resident	0.82*	0.82*	0.82*	1

## Discussion

In 2017, the American Academy of Periodontology and the European Federation of Periodontology provided a new diagnostic framework for periodontitis based on a multidimensional stage and grade system^4^. The stage related to the severity and extent of periodontitis should be determined using the clinical attachment loss (CAL), or radiographic bone loss (RBL) if the CAL is not available^4^. The stages from one to three are able to be determined among four stages with CAL or RBL^4^. This grading also allowed us to assess the rate of periodontitis progression, which is mainly determined by primary criteria consisting of direct and indirect evidence of progression^4^. The RBL evaluation is a highly valuable tool in assessing periodontal health^4,23–25^. Panoramic radiographs and intra-oral periapical radiographs have been mainly used for RBL evaluation. However, panoramic radiographs are the best modality to screen the lesions of the whole jaw. In addition, a previous study demonstrated a high level of agreement between intra-oral periapical and panoramic radiograph readings of distances between the CEJ level and the PBL as well as the proportional values in relation to the root length^26^. Manual measurement of the RBL of all teeth in a panoramic radiograph is also very time-consuming and labor-intensive. Therefore, an easy and automated method is essential to evaluate the RBL and accurately stage the periodontitis on the panoramic radiographs. For the first time, we developed an automatic method for diagnosing periodontal bone loss on dental panoramic radiographs to stage the periodontitis according to the new criteria proposed at the 2017 World Workshop^4^. As far as we know, no previous studies have quantitatively and automatically staged periodontitis using dental panoramic radiographs.

A recent CAD that was based on deep learning, which is a subset of machine learning, used the whole image directly without the best-feature representation by automatically extracting the relevant features during training^27^. The deep CNN was one of the most established algorithms for deep learning. In this study, a novel deep learning hybrid framework was proposed to automatically detect and classify the periodontal bone loss. We used a modified CNN from the Mask R-CNN to detect the PBL, the CEJL, and the teeth and implants. The R-CNN included two stages to identify a number of region of interests (ROIs) in the form of bounding-boxes, and to extract features from each ROI for classification^28^. The Mask R-CNN was an extension of the Faster R-CNN, after adding a branch that predicts the object mask in parallel with the existing branch for bounding box recognition^28^. In previous studies, a CNN based on VGG-19 backbone was used for feature extraction to diagnose periodontally-compromised teeth on periapical images^20^. In addition, a CNN using transfer learning was developed to improve the detection accuracy of periodontal bone loss on the panoramic radiographs^22^.

In this study, the radiographic bone level (or the CEJ level) was detected as a simple structure for the whole jaw on the panoramic radiographs using the CNN. We defined the partial oral cavity, enclosed by the periodontal bone levels of the maxilla and mandible, as one simple structure for the whole jaw. This structure decreased the complexity of the alveolar bone destruction patterns of the tooth. These patterns showed various destructive structures (aspects), such as horizontal bone loss, vertical/angular defects, osseous craters, and furcation involvement^29^. We also applied a similar process to detect the CEJ levels of the teeth as one structure at the maxilla and the mandible, respectively. When detecting the periodontal bone level, larger errors mainly occurred at both lateral sides of the enclosed oral cavity. However, these sides were irrelevant areas for determining the true periodontal bone level.

The detection accuracies of the dice coefficient were 0.93, 0.91, and 0.91 for the periodontal bone level, the CEJ level, and the teeth, respectively. In a previous study, a CNN using 12,179 panoramic radiographs showed an F1 score (dice coefficient) of 0.75 for direct detection of periodontal bone loss areas^22^. When the data for training the CNN are insufficient, the CNN model learns statistical regularity that is specific to the training set, and shows low accuracy for a new data set^30^. The best solution for this overfitting problem is to use a large amount of training data for each disease. However, a large dataset with exact annotations by specialists in medical imaging is not always available. Despite our relatively small amount of training data in deep learning, we still could achieve high detection performance of the periodontal bone level (or the CEJ level) by simplifying the complexity of the bone destruction patterns from periodontitis.

We automatically classified the periodontal bone loss of the individual tooth in order to stage the periodontitis based on the percentage rate analysis using conventional CAD processing. The percentage rate of the radiographic bone loss (RBL) for each tooth (implant) was determined directly by calculating the ratio of the intersection length of the periodontal bone level and that of the CEJ level for each tooth. The tooth intersection lengths are defined by the distances from the root apex point of the tooth to two intersection points of the tooth’s (implant) long-axis with the periodontal bone, and the CEJ levels (the fixture top level for implant). The long-axis orientation of the tooth, or the implant, was determined by applying the principal axes of inertia to their boundary images.

The mean absolute difference between the periodontitis staging performed by the automatic method and by the radiologists was **0.25** overall for the teeth of the whole jaw. The classification accuracy for the incisors and molars was lower than that of the canines and premolars, because the incisor roots were blurred by their overlapping with the vertebrae. The mesial and distal roots of the molars sometimes showed different periodontal bone levels with each other. The Pearson correlation coefficient between the automatic method and the radiologists was **0.73** overall for the whole jaw. The intraclass correlation coefficient was **0.91** overall for the whole jaw. Generally, the correlations between the automatic method and the radiologists were higher than those between radiologists themselves. This correlation was highest between the automatic method and the radiologist with the longest experience. Therefore, the automatic classification of periodontal bone loss for staging periodontitis had high accuracy and excellent diagnostic reliability.

Our method used the percentage rate of periodontal bone loss to automatically stage the periodontitis of the entire jaw according to the new criteria proposed at the 2017 World Workshop^4^. We achieved high detection performance of the periodontal bone level (or the CEJ level) by simplifying the complexity of the bone destruction patterns from periodontitis using deep learning. There was also high classification performance for automatic periodontitis staging using conventional CAD processing. A novel hybrid framework that combined deep learning architecture and conventional CAD approaches demonstrated high accuracy and excellent reliability in the automatic diagnosis of periodontal bone loss of individual teeth. We believe that this approach will help dental professionals to definitively diagnose and treat periodontitis. However, this method cannot provide the absolute value of the periodontal bone loss because of inconsistent image magnification and distortion in dental panoramic radiography. In future research, the performance of the developed method must be evaluated through interorganizational collaboration.

In conclusion, the developed method can help dental professionals to diagnose and monitor periodontitis systematically and precisely on panoramic radiographs. Therefore, it may substantially improve dental professionals’ performance with regard to the diagnosis and treatment of periodontitis.

## Materials and Methods

### Data preparation of dental panoramic radiographs

The panoramic radiographs of each patient were acquired in 2018 using a dental panoramic X-ray machine (Orthopantomograph OP 100D, Instumentarium corporation, Tuusula, Finland) at Seoul National University Dental Hospital. We prepared a total of 340 panoramic radiographs excluding the images of patients with primary or mixed dentition. The panoramic radiographs were collected retrospectively after removing identifiable patient information. The study was approved by the Institutional Review Board (IRB) of Seoul National University Dental Hospital (ERI18001) with a waiver of informed consent. The data collection and all experiments were performed in accordance with the relevant guidelines and regulations.

We used 330, 115, and 73 images in the panoramic image dataset for detecting the periodontal bone level (PBL), the cementoenamel junction level (CEJL), and the teeth, respectively. The images were randomly separated into a training set (90%), and a test set (10%) before data augmentation. The training set was used for CNN training of detection, and the testing set was used to evaluate the final trained model. We performed data augmentation to increase the number of data for deep learning by modifying the images. The images were flipped horizontally, rotated, and translated, and their gray values were transformed by contrast-normalization. Therefore, the number of images was increased by 64 times that of the original amount. To evaluate the classification performance for staging the periodontitis, we used ten panoramic radiographs not used for detection.

### Detection of PBL and CEJL structures using deep CNN

Considering the complexity of the alveolar bone destruction patterns of the tooth, we annotated the PBL as one simple structure for the whole jaw on the panoramic radiograph. The oral and maxillofacial (OMF) radiologists manually delineated an area inside the partial oral cavity that was enclosed by the periodontal bone levels of the maxilla and mandible using a labeling software (LabelBox, Labelbox Inc, CA). Using a similar procedure, we annotated the CEJ level of the teeth (the fixture top level of implants) as one structure that included the crowns of the teeth and implants at the maxilla and the mandible, respectively. We also annotated the rough boundaries of the teeth and implants.

We used a modified CNN from the Mask R-CNN based on a feature pyramid network (FPN) and a ResNet101 backbone to detect the PBL, CEJL, and the teeth on the panoramic radiograph^28^. The CNN was implemented using the python language with Keras and TensorFlow libraries. We modified the input resolution from the original size of 1976 × 976 pixels to 1024 × 1024 pixels to improve the training efficiency. We then trained the network for 300 iterations at a learning rate of 0.001, batch size of two images and two stride size (which was processed using eight graphics processing units) (GeForce GTX 1080 Ti, Nvidia, Santaclara, CA).

After training, the CNN produced the segmentation mask of the anatomical structures for the input panoramic image. A binary image of the structure enclosed by the periodontal bone levels was produced using binarization of the segmentation mask area and the other remaining area. The periodontal bone levels were then detected by extracting the edge of the binary image (Fig. 2a–e). The same process was applied to the detection of the CEJ level (Fig. 2f–j), the teeth and the implants from their segmentation mask (Fig. 2k–o).

### Classification of the periodontal bone loss by the percentage rate analysis

The principal axis of the tooth or the implant was determined by applying the principal axes of inertia to their boundary images^31–33^. Two principal axes directions, calculated by geometrical moments, defined the major axis of the maximum moment of inertia and the minor axis of the minimum moment of inertia. The minor axis direction was determined directly as the long-axis orientation of the tooth or the implant (Fig. 3a–e). We then found two intersection points of the tooth (implant) long-axis with the periodontal bone level and the CEJ level (the fixture top level for implant) detected by the CNN. We calculated the two tooth intersection lengths, which were the distances between those points and the root apex point of the tooth.

We defined the percentage rate of the radiographic bone loss (RBL) for the tooth (implant) as the ratio of the intersection length of the periodontal bone level and the other CEJ level (the fixture top level for implant) (Fig. 3f–j). Based on the percentage rate, we automatically classified the periodontal bone loss of the tooth in order to stage the periodontitis according to the new criteria proposed at the 2017 World Workshop on the Classification of Periodontal and Peri-implant Diseases and Conditions^4^ (Fig. 3k–o). The classification criteria for staging the periodontitis based on the RBL of the tooth were as follows: (1) If the RBL was <15% (in the coronal third of the root), the periodontitis was classified as the stage one; (2) if the RBL was between 15% and 33% (in the coronal third of the root), it was classified as the stage two; and (3) if the RBL was >33% (extending to the middle third of the root and beyond), then it was classified as the stage three^4^.

### Evaluation of detection and classification performance

In order to evaluate the CNN detection performance, we measured the following: a pixel accuracy (PA), the percentage of correctly identified pixels (TP/(TP + FN)), the dice coefficient (F1 score) ($$2|{S}_{gt}{\cap }^{}{S}_{det}|/(|{S}_{gt}|+|{S}_{det}|)$$), the Jaccard index ($$|{S}_{gt}{\cap }^{}{S}_{det}|/$$$$|{S}_{gt}{\cup }^{}{S}_{det}|$$), the similarity between the ground-truth ($${S}_{gt}$$) and the detected ($${S}_{det}$$) structures^34–36^. To evaluate the classification performance for periodontitis staging, we compared the automatically determined stages (by the percentage rate analysis of RBL) to those made by three OMF radiologists (including a resident with three-years of experience, a fellow with five-years of experience and a professor with ten-years of experience). We compared the mean absolute difference between the stage values using the developed method and the radiologists’ diagnoses using the ANOVA test. We also calculated Pearson correlation coefficients (PCC) and intraclass correlation coefficients (ICC) between the stage values using SPSS software^37,38^ (SPSS, SPSS Inc., Chicago, IL, USA).

## Data Availability

The dental panoramic radiographs in dataset used to develop and analyze the findings of this study are not publicly available due to the restriction by the Institutional Review Board (IRB) of Seoul National University Dental Hospital in order to protect patients’ privacy.

## References

[1] Tonetti MS, Jepsen S, Jin LJ, Otomo-Corgel J (2017). Impact of the global burden of periodontal diseases on health, nutrition and wellbeing of mankind: A call for global action. J. Clin. Periodontol..

[2] Lang NP, Bartold PM (2018). Periodontal health. J. Periodontol..

[3] Caton JG (2018). A new classification scheme for periodontal and peri-implant diseases and conditions - Introduction and key changes from the 1999 classification. J. Periodontol..

[4] Tonetti MS, Greenwell H, Kornman KS (2018). Staging and grading of periodontitis: Framework and proposal of a new classification and case definition. J. Periodontol..

[5] Garnick JJ, Silverstein L (2000). Periodontal probing: Probe tip diameter. J. Periodontol..

[6] Keagle JG, Garnick JJ, Searle JR, King GE, Morse PK (1989). Gingival Resistance to Probing Forces .1. Determination of Optimal Probe Diameter. J. Periodontol..

[7] Trombelli L, Farina R, Silva CO, Tatakis DN (2018). Plaque-induced gingivitis: Case definition and diagnostic considerations. J. Periodontol..

[8] Wang CW (2016). A benchmark for comparison of dental radiography analysis algorithms. Med. Image Anal..

[9] Schmidhuber J (2015). Deep learning in neural networks: An overview. Neural Networks.

[10] Lee H, Park M, Kim J (2017). Cephalometric landmark detection in dental x-ray images using convolutional neural networks. Medical Imaging 2017: Computer-aided Diagnosis.

[11] Ronneberger O, Fischer P, Brox T. Dental X-ray image segmentation using a U-shaped Deep Convolutional network. *International Symposium on Biomedical Imaging*, (2015).

[12] Miki Y (2017). Classification of teeth in cone-beam CT using deep convolutional neural network. Comput. Biol. Med..

[13] Jader, G. *et al*. JMKML. Deep instance segmentation of teeth in panoramic X-ray images. *Conference on Graphics*, *Patterns and Images (SIBGRAPI**)*, 400-407 (2018).

[14] Ben, A. R., Ridha, E. & Mourad, Z. Detection and classification of dental caries in x-ray images using deep neural networks. *International Conference on Software Engineering Advances (ICSEA)*, 223–227 (2016).

[15] Srivastava, M. M., Kumar, P., Pradhan, L. & Varadarajan, S. Detection of Tooth caries in Bitewing Radiographs using Deep Learning. https://arxiv.org/abs/1711.07312 (2017).

[16] Choi J, Eun H, Kim C (2018). Boosting Proximal Dental Caries Detection via Combination of Variational Methods and Convolutional Neural Network. J. Signal Process. Sys..

[17] Lee JH, Kim DH, Jeong SN, Choi SH (2018). Detection and diagnosis of dental caries using a deep learning-based convolutional neural network algorithm. J. Dent..

[18] Hiraiwa T (2019). A deep-learning artificial intelligence system for assessment of root morphology of the mandibular first molar on panoramic radiography. Dentomaxillofac. Rad..

[19] Murata M (2018). Deep-learning classification using convolutional neural network for evaluation of maxillary sinusitis on panoramic radiography. Oral Radiol..

[20] Lee J-H, Kim D-h, Jeong S-N, Choi S-H (2018). Diagnosis and prediction of periodontally compromised teeth using a deep learning-based convolutional neural network algorithm. J. Periodontal Implant Sci..

[21] Krois J (2019). Deep Learning for the Radiographic Detection of periodontal Bone Loss. Sci. Rep..

[22] Kim J, Lee H-S, Song I-S, Jung K-H (2019). DeNTNet: Deep Neural Transfer Network for the detection of periodontal bone loss using panoramic dental radiographs. Sci. Rep..

[23] Albandar JM, Abbas DK (1986). Radiographic Quantification of Alveolar Bone Level Changes - Comparison of 3 Currently Used Methods. J. Clin. Periodontol..

[24] Bjorn H, Holmberg K (1966). Radiographic determination of periodontal bone destruction in epidemiological research. Odontol. Rev..

[25] Lang NP, Hill RW (1977). Radiographs in Periodontics. J. Clin. Periodontol..

[26] Persson RE (2003). Comparison between panoramic and intra-oral radiographs for the assessment of alveolar bone levels in a periodontal maintenance population. J. Clin. Periodontol..

[27] Yamashita R, Nishio M, Do RKG, Togashi K (2018). Convolutional neural networks: an overview and application in radiology. Insights Imaging.

[28] He KM, Gkioxari G, Dollar P, Girshick R (2020). Mask R-CNN. IEEE T. Pattern Anal..

[29] Ozcan G, Sekerci AE (2017). Classification of alveolar bone destruction patterns on maxillary molars by using cone-beam computed tomography. Niger. J. Clin. Pract..

[30] Shorten C, Khoshgoftaar TM (2019). A survey on Image Data Augmentation for Deep Learning. J. Big Data.

[31] Kim DS (2013). Principal direction of inertia for 3D trajectories from patient-specific TMJ movement. Comput. Biol. Med..

[32] Yi WJ, Heo MS, Lee SS, Choi SC, Huh KH (2007). Comparison of trabecular bone anisotropies based on fractal dimensions and mean intercept length determined by principal axes of inertia. Med. Biol. Eng. Comput..

[33] Yi WJ (2007). Direct measurement of trabecular bone anisotropy using directional fractal dimension and principal axes of inertia. Oral Surg. Oral Med. Oral Pathol. Oral Radiol. Endod..

[34] Polak M, Zhang H, Pi MH (2009). An evaluation metric for image segmentation of multiple objects. Image Vision Comput..

[35] Seyedhosseini M, Tasdizen T (2016). Semantic Image Segmentation with Contextual Hierarchical Models. IEEE T. Pattern Anal..

[36] Zou KH (2004). Statistical validation of image segmentation quality based on a spatial overlap index. Acad. Radiol..

[37] Hunt RJ (1986). Percent agreement, Pearson’s correlation, and kappa as measures of inter-examiner reliability. J. Dent. Res..

[38] Muller R, Buttner P (1994). A critical discussion of intraclass correlation coefficients. Stat. Med..

